# Nothing in It

**Published:** 1880-06

**Authors:** 


THE *Philadelphia Medical Times* gives the following:

Some time since, a young assistant, desiring to be very painstaking, brought as the result of one night's watching of a very serious case nearly a quire of closely-written foolscap, of which the following is a sample:

"1.15 A. M. The patient is seen to slowly rise from his bed and seat himself upon the chamber, where he remains with much straining and looks of anguish for some minutes. 1.30 A. M. He has returned to bed. 1.35 A. M. Examined the pot. Nothing in it."

To which a note might be well appended, "Examined the assistant's head. Nothing in it."

